# Supplementary material for: Conveying tactile object characteristics through customized intracortical microstimulation of the human somatosensory cortex
Source: Nat Commun. 2025 May 1;16:4017. doi: 10.1038/s41467-025-58616-6 (PMC12046030; doi:10.1038/s41467-025-58616-6)
Supplement: Supplementary file 1 — Supplementary Information [file 41467_2025_58616_MOESM1_ESM.pdf]

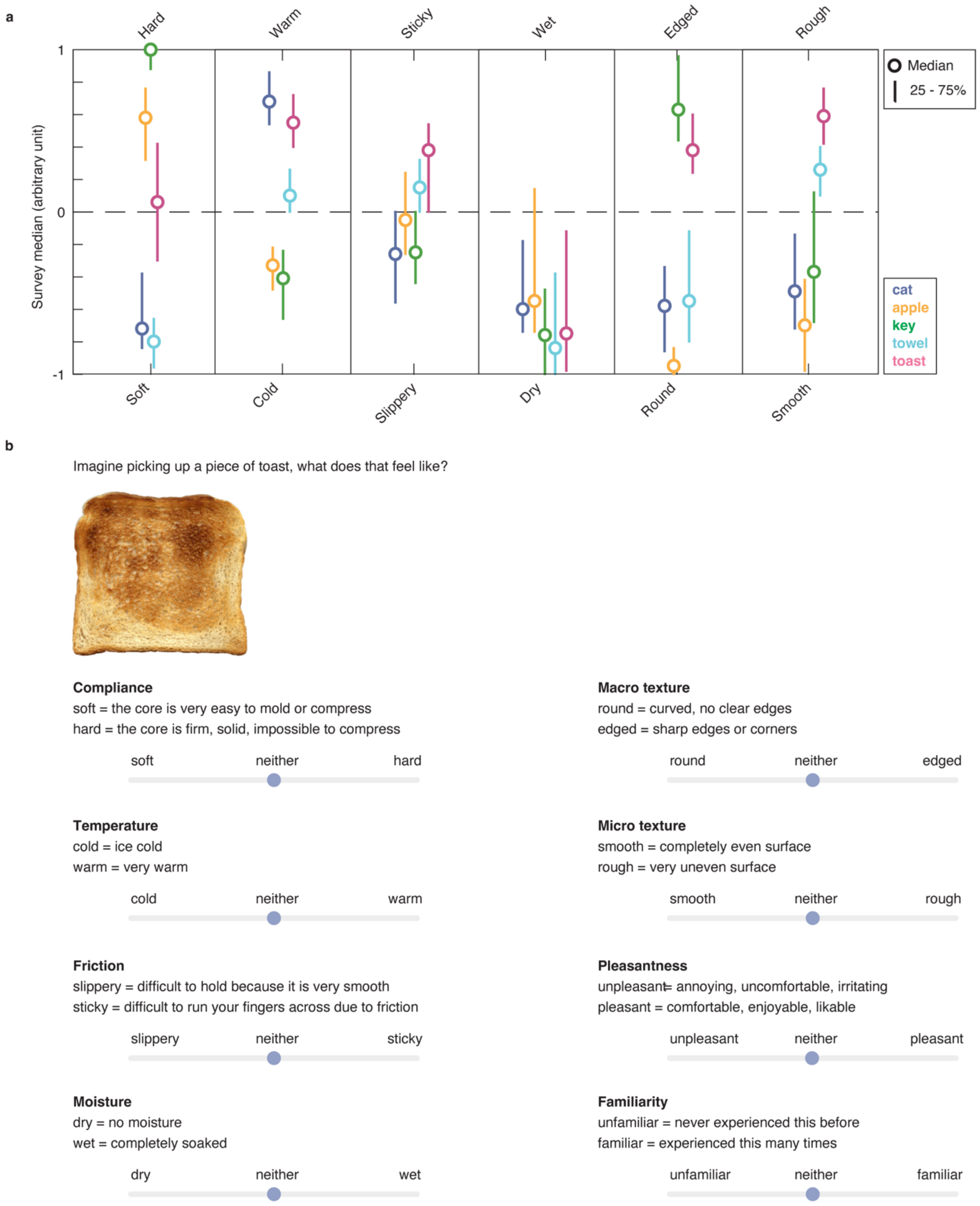

Figure S1. *Tactile quality survey results of 34 people with intact somatosensation.* ***a.*** *Median levels with 25-75 percentile ranges of compliance, temperature, friction, moisture, micro and macro structure ascribed to each object according during the object quality survey. Source data are provided as a Source Data file.* ***b.*** *Example screenshot of a single item in the online survey. A total of 30 objects were presented, including an apple, banana peel, book, cat, cinnamon roll, glass, pair of glasses, pair of gloves, hammer, hand, ice cube, electric toothbrush, rock, key, knife, needle, orange,*

pencil, smartphone, rabbit, boiled rice, pair of scissors, pair of socks, used sponge, sweater, teddy bear, piece of toast, roll of toilet paper, towel and a wig. Each object was associated with a specific action: pick up, pet, shake, or grab. Participants were asked to rate the compliance, temperature, friction, moisture, macro texture, micro texture, pleasantness, and familiarity of these imagined object interactions. The toast image was made by Rainer Zenz and sourced from Commons.wikimedia.org (license: creativecommons.org/licenses/by-sa/3.0). No changes were made to the image.

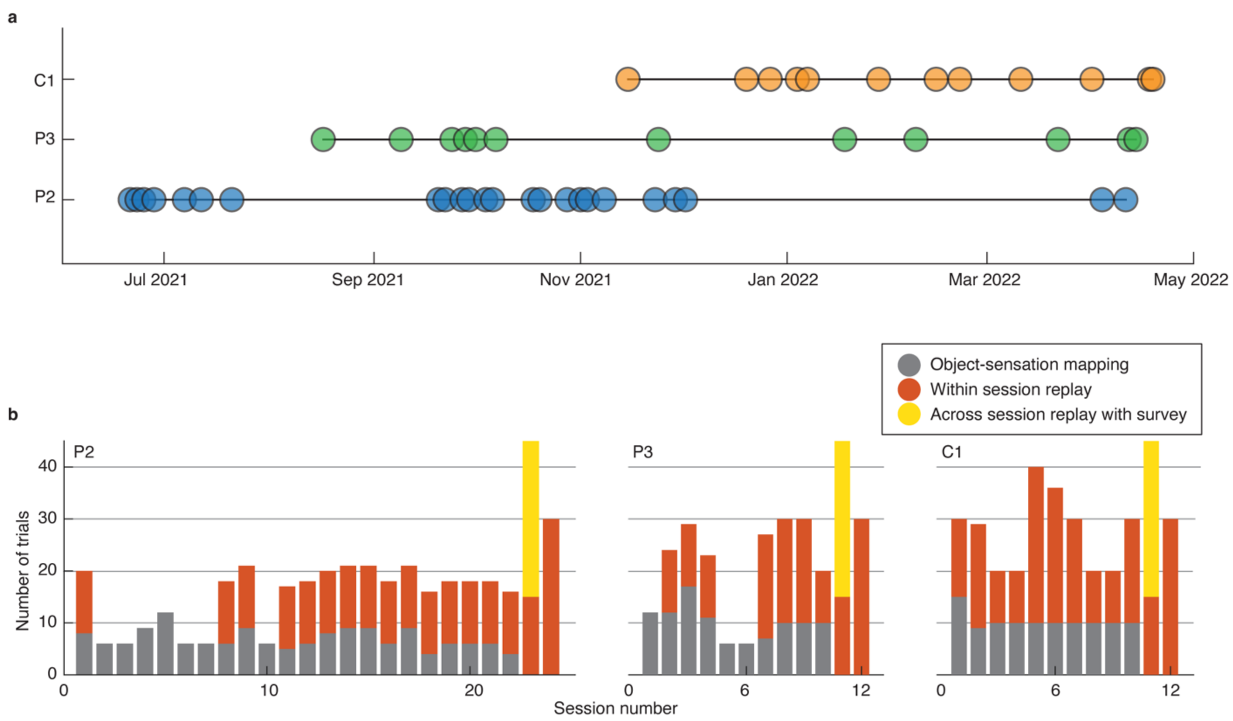

Figure S2. *Experiment timeline.* ***a.*** *Dates of individual sessions for each participant.* ***b.*** *Overview of the number of collected trials during each session, for each participant. There are three unique trial types: object-sensation mapping trials, replay trials and replay trials with a sensory survey. Source data of both a and b figure panels are provided as a Source Data file.*

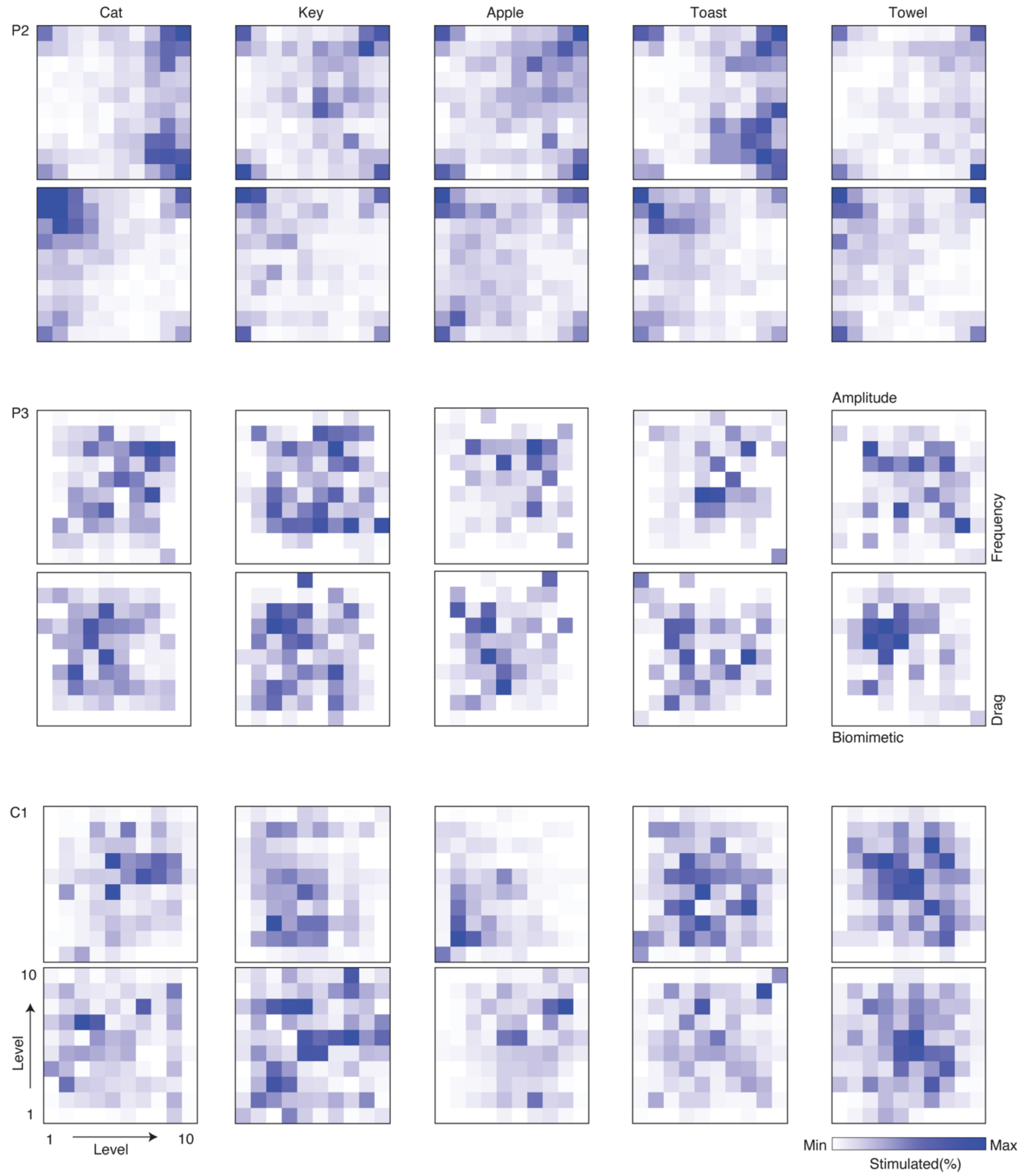

Figure S3. *Total percentage of time across all repetitions of each object spent on a certain stimulation setting for each participant. Parameter values increase from left (level 1) to right (level 10), and bottom (level 1) to top (level 10). Dark blue areas show "hotspots" within the parameter spaces where the participant spent most exploration time for a certain object. Source data are provided as a Source Data file.*

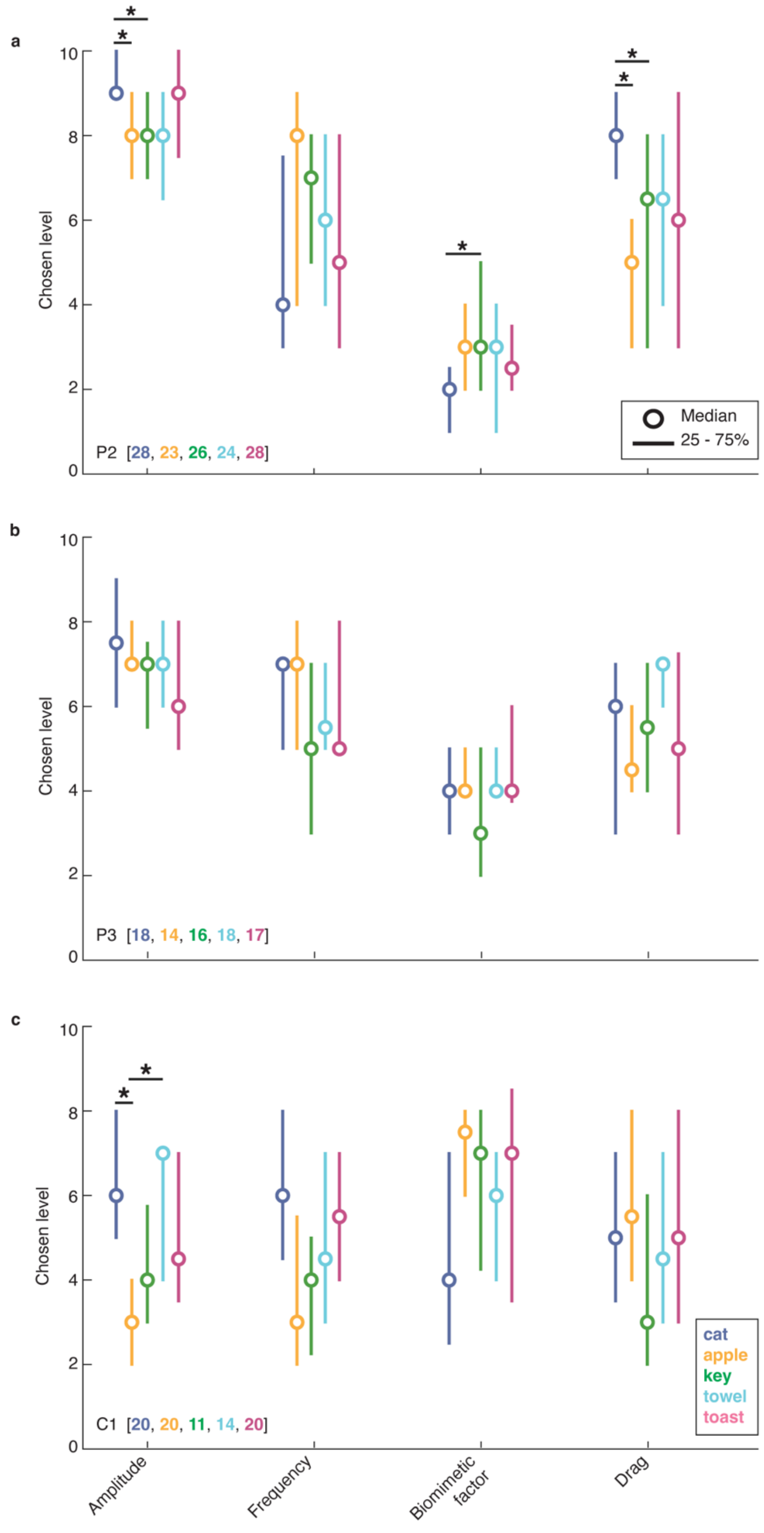

Figure S4. *Object-specific stimulus parameter choices for each participant.* ***a.*** *Median and 25-75 percentile ranges of each stimulus parameter per object. The data include only those trials that had a normalized satisfaction score of at least 50 out of 100. The number of repetitions per object is indicated in brackets for each object and participant. For participant P2, the amplitude was significantly (*) different between a cat and apple (p = 0.004, one-sided Kolmogorov-Smirnov test*

Bonferroni corrected at α = 0.005, D = 0.48) as well as a cat and key ($p = 0.008^{-1}$, D = 0.52). The biomimetic factor was significantly different for a cat and key (p = 0.002, D = 0.48), and the drag was significantly different for a cat and apple ($p = 0.001^{-1}$, D = 0.59). ***b.*** *Same as a., but for participant P3. No significant differences were found (Table S1).* **c.** *Same as b., but for participant C1. The amplitude was significantly (*) different for a cat and apple ($p = 0.003^{-1}$, D = 0.64). Source data of figure panels a-c are provided as a Source Data file.*

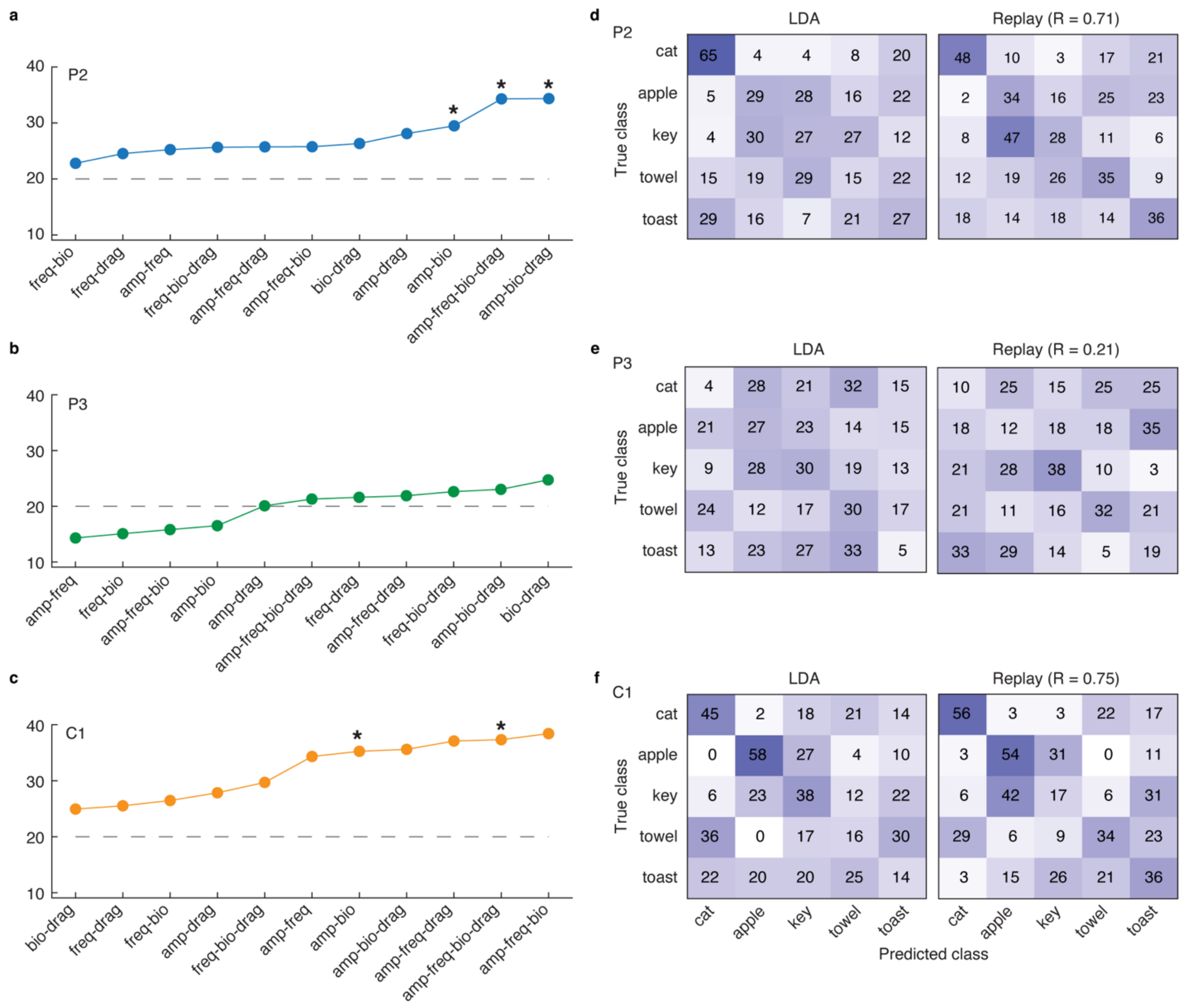

Figure S5. *LDA results per participant.* ***a.*** *Mean LDA performance on all different combinations of stimulus parameters across 100 bootstrapped repetitions. In addition to the significant performance on all four stimulus parameters, a significant (*) performance was found by including amplitude and biomimetic factor information (p = 0.002, one-sided permutation test with 1000 permutations without replacement, Bonferroni corrected at α = 0.003, accuracy = 29%), and amplitude, biomimetic factor information and drag (p = 0.002, accuracy = 34%).* ***b.*** *Same as d., but for participant P3. No combination of parameters led to a significant classification performance.* ***c.*** *Same as e., but for participant C1. Similarly to P2, a significant (*) classification performance was found by combining amplitude and biomimetic factor information (p = 0.002, accuracy = 35%) in addition to combining all four stimulus parameters.* ***d.*** *Full confusion matrix for the LDA classifier and participant performance of P2. The R-value indicates the correlation between the participant and LDA performances. The numbers in each row indicate the percentage of times objects were correctly or incorrectly classified: for example, cat sensations that were classified by the LDA (left) or participant (right) as belonging to a cat, apple, key, towel or toast.* ***e.*** *Same as a., but for participant P3,* ***f.*** *Same as b., but for participant C1. There was a strong positive correlation between the perceptual replay and stimulus-parameter-based LDA performances of participant P2 (Pearson's r(23) = 0.71, p = 0.001^{-1}) and C1 (r(23) = 0.75, p = 0.001^{-2}), and a low positive correlation for P3 (r(23) = 0.21, p = 0.314)). Source data of figure panels a-f are provided as a Source Data file.*

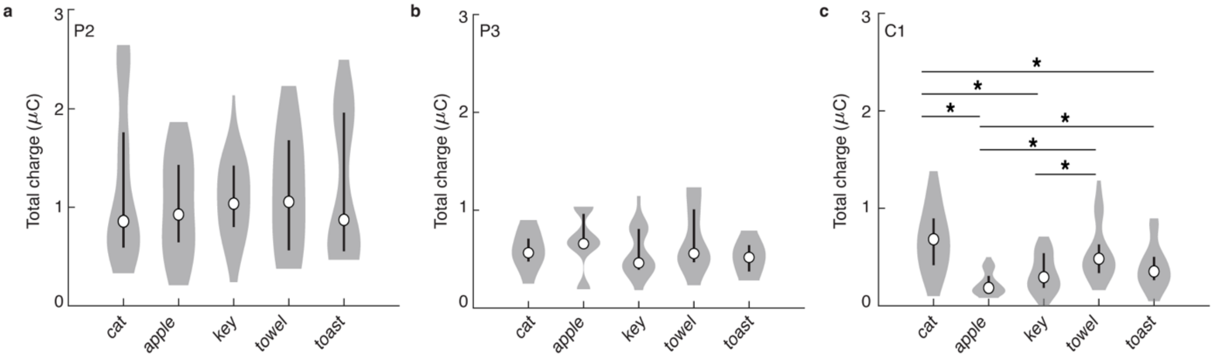

Figure S6. *Normalized total charge of the object-specific stimulus choices of participant* ***a.*** *P2 (29 cat, 44 apple, 36 key, 43 towel, 28 toast sensations),* ***b.*** *P3 (18 cat, 11 apple, 23 key, 17 towel and 15 toast sensations) and* ***c.*** *C1 (36 cat, 35 apple, 35 key, 35 towel and 39 toast sensations) in the replay task. No significant differences in mean charge across all electrodes were found between the object-specific stimulation trains of P2 (p = 0.768, two-sided Kruskal-Wallis test, α = 0.025, $\chi^2(4) = 1.83$) and P3 (p = 0.238, $\chi^2(4) = 5.52$). However, significant differences (*) were found in the mean charge between the object-specific stimulation trains created by C1 (p = 0.004^{-8}, χ2(4) = 54.77). C1 created higher charge (one-sided Kolmogorov-Smirnov test, Bonferroni corrected at* α = 0.005*) stimulation trains for a cat compared to that of an apple (p = 0.005^{-6}, D = 0.72), key (p = 0.001^{-2}, D = 0.56) or toast (p = 0.005^{-2}, D = 0.46). Similarly, the charge created for a towel was higher than that of an apple (p = 0.007^{-4}, D = 0.63) and key (p = 0.003, D = 0.41), and the charge created for toast was bigger than that for an apple (p = 0.002^{-1}, D = 0.48). To check whether the total charge per electrode could explain the significant classification performances of P2 and C1, an additional LDA classifier was trained using 10-fold cross validation based on the total charge per electrode alone. This LDA classifier reached a performance of 20% (p = 0.511, permutation test, 1000 permutations without replacement,* α = 0.05*) for P2, 18% (p = 0.743) for P3 and 34% (p = 0.001) for C1. These significant differences in total charge maybe due to C1's preferred use of automatic stimulation during the object-sensation mapping task. Whereas P2 and P3 used almost exclusively automatic stimulation, evoking an identical cursor movement across each object, C1 manually explored the objects to evoke stimulation. Moreover, C1 explicitly reported using different manual exploration strategies for each object, e.g., stroking a cat along its back and drawing fast circles across a towel. The differences in cursor velocity per object will influence the result of the biomimetic factor differently, potentially enhancing differences in total charge between objects. Source data of figure panels a-c are provided as a Source Data file.*

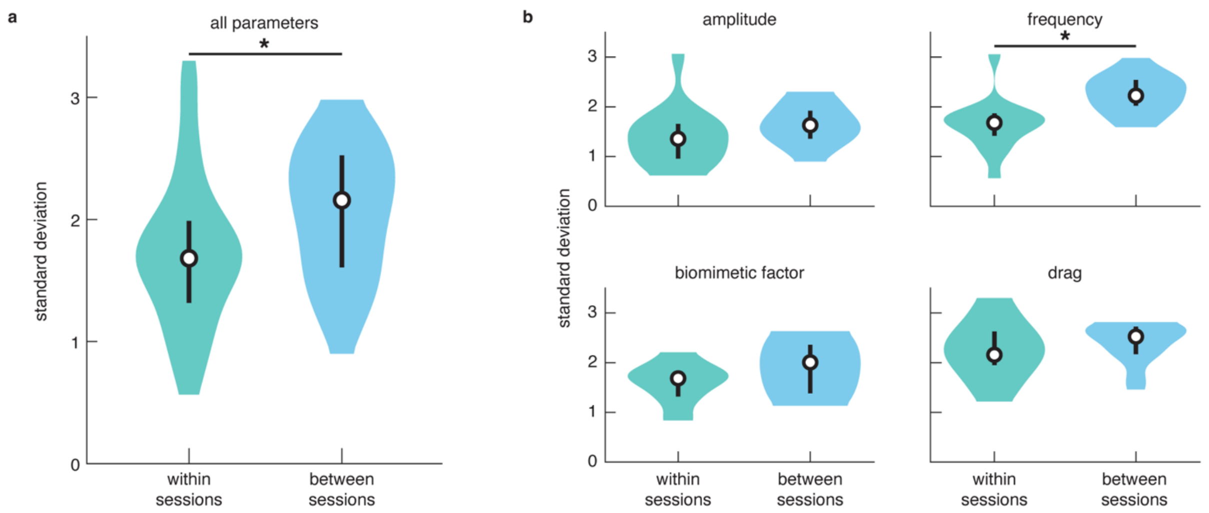

Figure S7. *Variation of stimulus profiles within and across test sessions.* ***a.*** *The distribution of object-specific standard deviations across all parameters and participants was significantly (*) lower within sessions than across sessions (p = 0.002, one-sided Kruskal-Wallis test, α = 0.05, χ2(1) = 9.95, 60 samples within and 60 samples between sessions).* ***b.*** *Same as in a., but shown for each individual stimulus parameter. The significant difference (*) in within and across session parameter variation was explained by a significantly larger variation in the frequency parameter across sessions compared to within sessions ($p = 0.002^{-1}$, one-sided Kolmogorov-Smirnov test, Bonferroni corrected at α = 0.01, D = 0.73, 15 samples within and 15 samples between sessions). Source data of figure panels a and b are provided as a Source Data file.*

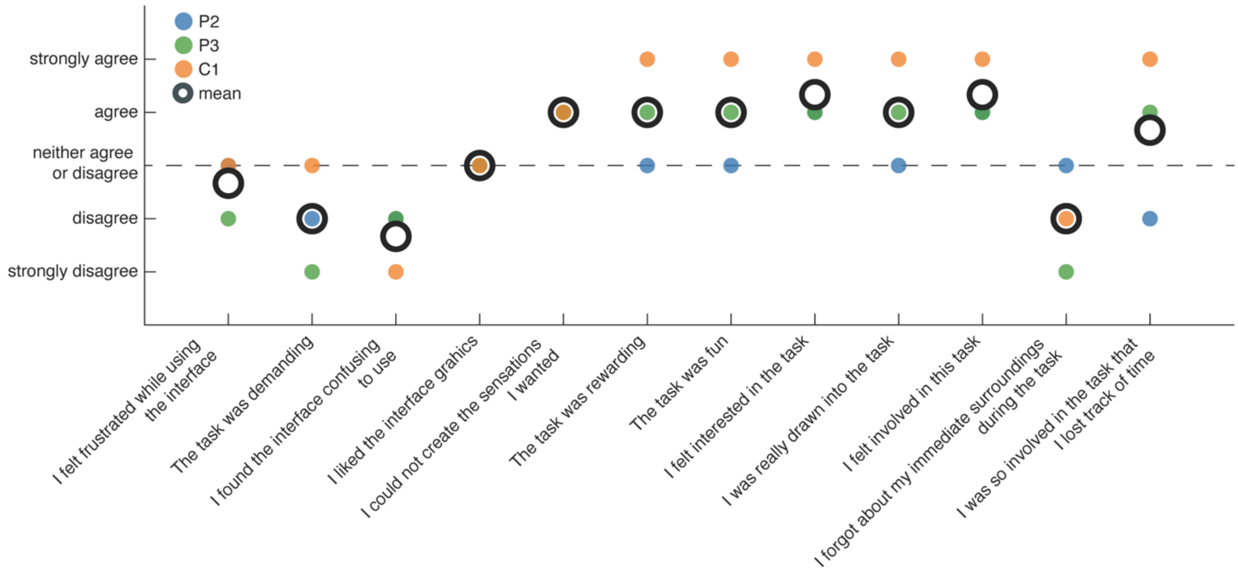

Figure S8. *Participant engagement survey. The response of each participant is represented by a colored dot. The mean response across participants is highlighted by a black circle. Overall, participants were positive about their experiences using the tablet interface. Source data are provided as a Source Data file.*

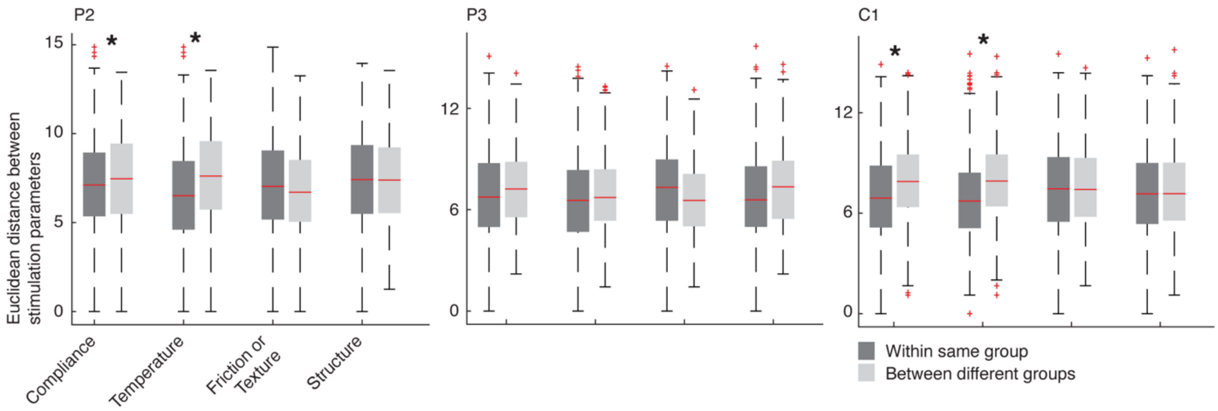

Figure S9. *Euclidean distance between chosen stimulation parameters for objects with similar (same group) and different (different group) tactile characteristics. For example, based on the compliance ratings of the object quality survey, cats and towels were considered soft, whereas toast, apples and keys were hard. Across all participants, the chosen stimulation parameters for objects with similar compliance or temperature were significantly more similar to each other than those of objects with different compliance or temperature. For P2 and C1, stimulus parameters within the same compliance level were significantly more similar to each other than between different levels of compliance (P2: $p = 0.001$, Wilcoxon rank sum, Bonferroni-corrected $\alpha = 0.013$, $z = -3.025$, 2631 same and 1564 different group samples; C1: $p = 0.002^{-17}$, $z = -9.181$, 4602 same and 2542 different group samples). Similarly, stimulus parameters within the same temperature level were significantly more similar to each other than between different levels of temperature (P2: $p = 0.002^{-7}$, $z = -6.248$, 2885 same and 1500 different group samples; C1: $p = 0.001^{-15}$, $z = -8.756$, 2520 same and 4686 different group samples). No significant differences were found for participant P3. Source data are provided as a Source Data file.*

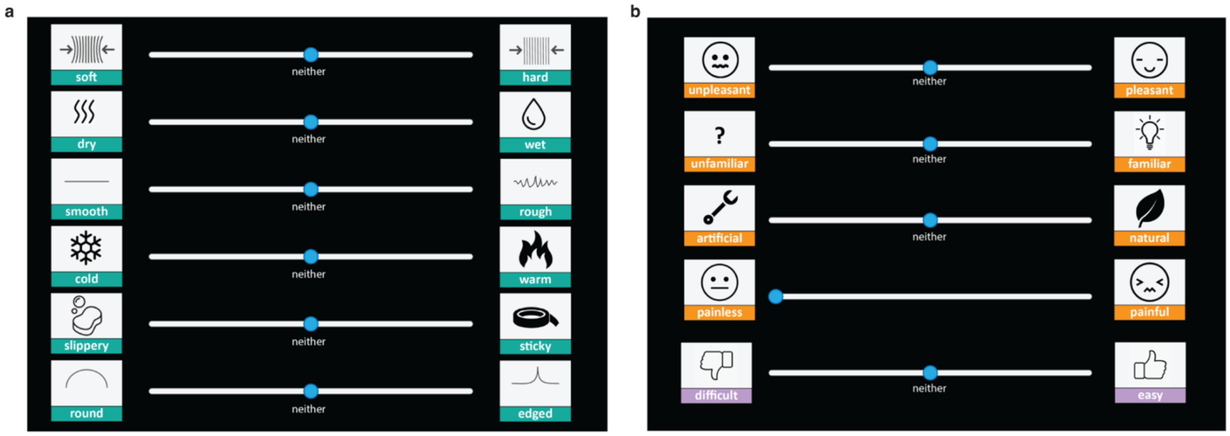

Figure S10. *Screenshot of the tablet interface with the* ***a.*** *tactile and* ***b.*** *affective surveys. Participants could 'flip' the icon images to reveal a more elaborative description (identical to those used in Fig. S1) of the indicated tactile characteristic. Each slider (with the exception of the 'pain' one) started from a default position in the middle, indicating that this tactile characteristic was not experienced. Participants could slide it to the left or right to report their experienced sensations. The image icons for the soft/hard, smooth/rough, round/edged, unpleasant, unfamiliar and painless dimensions were made in Adobe Illustrator. The familiar, artificial/natural and difficult/easy icons were sourced from Microsoft PowerPoint. The wet, cold/warm, pleasant/painful icons were made by Good Ware, Freepik, and Chanut, respectively, and sourced from Flaticon.com. The dry icon was sourced from Icons8.com, the slippery icon from Cleanpng.com, and the sticky icon was made by Brickclay and sourced from Thenounproject.com.*

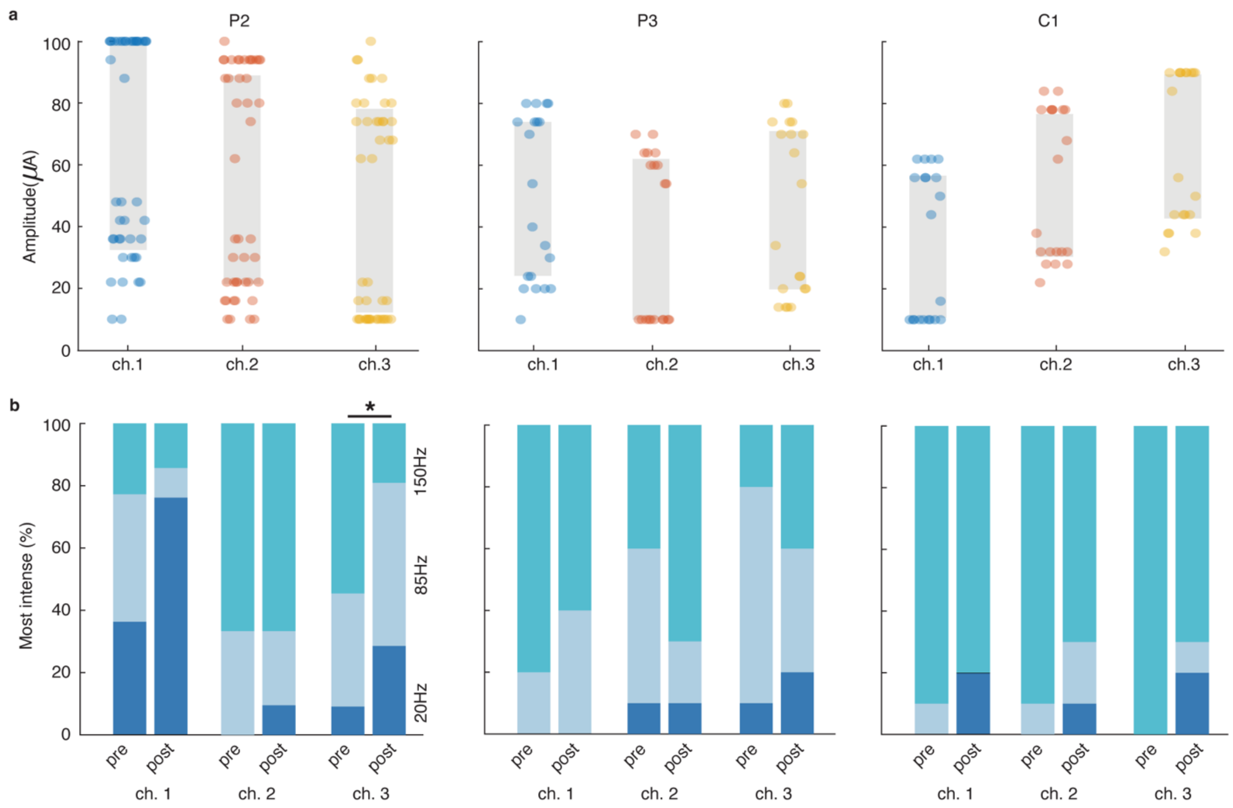

Figure S11. *Pre-test parameter selections across 22 repetitions per electrode for participant P2 and 10 repetitions per electrode for participants P3 and C1.* ***a.*** *Range of amplitudes used across all experimental test sessions per participant for the object-sensation mapping task. The grey rectangles indicate the mean minimum and maximum amplitudes per electrode. Clear differences were observed in the perceived intensity across the three stimulation electrodes. At the start of a session, participant P2 matched the electrode intensities with an average difference in amplitude of +11±10 μA between electrodes 1 and 2, and +20±13 μA between electrodes 1 and 3, P3 with a difference of +14±9 μA between electrodes 1 and 2, and +4±1 3μA between electrodes 1 and 3, and C1 with a difference of -20±4 μA between electrodes 1 and 2 and -32±6 μA between electrodes 1 and 3. We checked whether the results of this intensity matching procedure were consistent throughout a session and found no significant differences in the selected amplitude ratios between the start and end of a session (Kolmogorov-Smirnov test with P2: ch. 1-2 p = 0.423, ch. 1-3 p = 0.772; P3: ch. 1-2 p = 0.975, ch. 1-3 p = 1; C1: ch. 1-2 p = 0.675, ch. 1-3 p = 0.313).* ***b.*** *Total number of times that participants rated a low (20 Hz), medium (85 Hz) or high (100 Hz) stimulus frequency as most intense on a specific electrode. There was considerable variation across the intensity reports for all participants, e.g., although C1 rated a 150 Hz stimulus to be most intense on 80-85% of all trials on each electrode, he still selected a 20 Hz stimulus as most intense for 5-10% of the trials, and an 85 Hz stimulus as most intense on 5-15% of the trials. Except for electrode 3 of participant P2, the frequency and electrode specific differences in intensity were consistent at the start and end of a session (Wilcoxon signed rank test with P2: ch. 1 p = 0.025, ch. 2 p = 0.843, ch. 3 = .013*; P3: ch. 1 p = 0.366, ch. 2 p = 0.269, ch. 3 p = 0.705; C1: ch. 1 p = 0.503, ch. 2 p = 0.278 ch. 3 p = 0.078). Source data of figure panels a and b are provided as a Source Data file.*

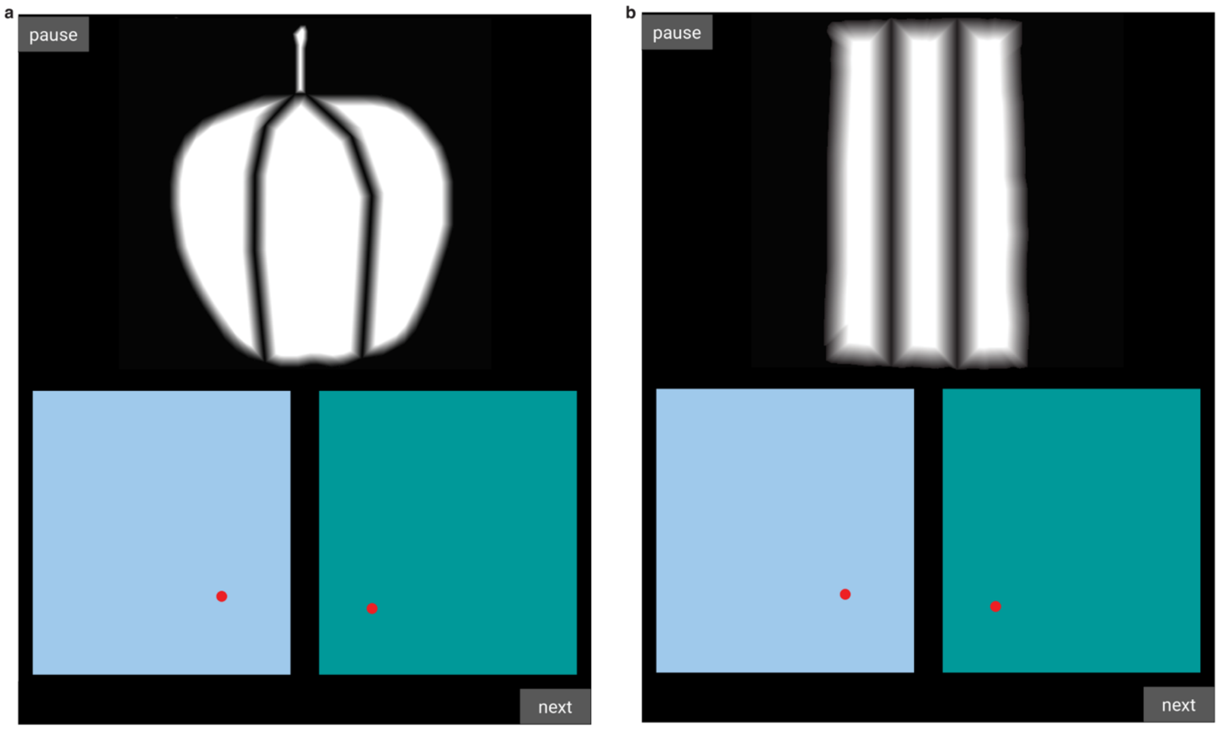

Figure S12. *Object masks for **a**. a hard (apple) and **b**. a soft (towel) object. The gradients created a pixel value for the on- and offset of each of the three electrodes "receptive field" on the displayed object. These object masks were used during the object-sensation mapping task. During the replay task, where the object was represented as a grey rectangle, identical gradients were used, albeit projected onto a rectangle rather than the original image shape.*

Table S1. Two-sided Kruskal-Wallis test results of differences between individual stimulus parameters across objects. After Bonferroni correction, the significance level was set to 0.006. Asterisks denote statistically significant results.

Index	Participant	Variable	p-value	Test statistic
1	P2	amplitude	$.001^{-1}$*	$\chi^2(4) = 23.16$
2	P2	frequency	.103	$\chi^2(4) = 7.72$
3	P2	biomimetic factor	.003*	$\chi^2(4) = 16.13$
4	P2	drag	.004*	$\chi^2(4) = 15.5$
5	P3	amplitude	.458	$\chi^2(4) = 3.63$
6	P3	frequency	.534	$\chi^2(4) = 3.14$
7	P3	biomimetic factor	.296	$\chi^2(4) = 4.92$
8	P3	drag	.137	$\chi^2(4) = 6.98$
9	C1	amplitude	$.002^{-1}$*	$\chi^2(4) = 21.86$
10	C1	frequency	.063	$\chi^2(4) = 8.92$
11	C1	biomimetic factor	.033	$\chi^2(4) = 10.48$
12	C1	drag	.295	$\chi^2(4) = 4.93$

Table S2. One-sided permutation test results (1000 permutations without replacement) of tactile quality specific individual classifiers. The significance level was set to 0.05. Asterisks denote statistically significant results.

Index	Participant	Variable	p-value
1	P2	compliance (63% accurate)	.009*
2	P2	temperature (72% accurate)	.000*
3	P2	friction/texture (55% accurate)	.184
5	P2	macro struct. (53% accurate)	.355
6	C1	compliance (71% accurate)	.001*
7	C1	temperature (76% accurate)	.000*
8	C1	friction/texture (63% accurate)	.072
10	C1	macro struct. (47% accurate)	.777
