# Supplementary material for: Conveying tactile object characteristics through customized intracortical microstimulation of the human somatosensory cortex
Source: Nat Commun. 2025 May 1;16:4017. doi: 10.1038/s41467-025-58616-6 (PMC12046030; doi:10.1038/s41467-025-58616-6)
Supplement: Supplementary file 2 — Reporting Summary [file 41467_2025_58616_MOESM2_ESM.pdf]

nature portfolio

Corresponding author(s): Robert Gaunt

Last updated by author(s): Jul 22, 2024

Reporting Summary

Nature Portfolio wishes to improve the reproducibility of the work that we publish. This form provides structure for consistency and transparency in reporting. For further information on Nature Portfolio policies, see our Editorial Policies and the Editorial Policy Checklist.

Statistics

For all statistical analyses, confirm that the following items are present in the figure legend, table legend, main text, or Methods section.

n/a	Confirmed	
☐	☒	The exact sample size (*n*) for each experimental group/condition, given as a discrete number and unit of measurement
☐	☒	A statement on whether measurements were taken from distinct samples or whether the same sample was measured repeatedly
☐	☒	The statistical test(s) used AND whether they are one- or two-sided<br>*Only common tests should be described solely by name; describe more complex techniques in the Methods section.*
☐	☒	A description of all covariates tested
☐	☒	A description of any assumptions or corrections, such as tests of normality and adjustment for multiple comparisons
☐	☒	A full description of the statistical parameters including central tendency (e.g. means) or other basic estimates (e.g. regression coefficient) AND variation (e.g. standard deviation) or associated estimates of uncertainty (e.g. confidence intervals)
☐	☒	For null hypothesis testing, the test statistic (e.g. *F*, *t*, *r*) with confidence intervals, effect sizes, degrees of freedom and *P* value noted<br>*Give P values as exact values whenever suitable.*
☒	☐	For Bayesian analysis, information on the choice of priors and Markov chain Monte Carlo settings
☒	☐	For hierarchical and complex designs, identification of the appropriate level for tests and full reporting of outcomes
☐	☒	Estimates of effect sizes (e.g. Cohen's *d*, Pearson's *r*), indicating how they were calculated

Our web collection on statistics for biologists contains articles on many of the points above.

Software and code

Policy information about availability of computer code

Data collection	Neural data were collected using NeuroPort Central software (Blackrock Microsystems, Inc.). These data, along with behavioral data, were formatted and saved for analysis using our own code toolbox in Python 3.7.6 and Matlab 2021a. The code used for data collection can be made available upon request to the study PI.
Data analysis	All data were analyzed using our own code in Matlab 2020b. Customized code used for analysis is available through Github at https://doi.org/10.5281/zenodo.14722512.

For manuscripts utilizing custom algorithms or software that are central to the research but not yet described in published literature, software must be made available to editors and reviewers. We strongly encourage code deposition in a community repository (e.g. GitHub). See the Nature Portfolio guidelines for submitting code & software for further information.

Data

Policy information about availability of data

All manuscripts must include a data availability statement. This statement should provide the following information, where applicable:

- Accession codes, unique identifiers, or web links for publicly available datasets
- A description of any restrictions on data availability
- For clinical datasets or third party data, please ensure that the statement adheres to our policy

The de-identified data that support the findings of this study are available under restricted access for participant privacy. Access can be obtained upon request to the study PI by an investigator who is prepared to securely handle data resulting from human research. The study PI will respond within 1 month after a request is

made. The data are shared via the Data Archive BRAIN Initiative (DABI) under project code JZITRHZ6X9WI at https://doi.org/10.18120/5b2x-2w11. Source data are provided with this paper.

Research involving human participants, their data, or biological material

Policy information about studies with human participants or human data. See also policy information about sex, gender (identity/presentation), and sexual orientation and race, ethnicity and racism.

Reporting on sex and gender	The study was conducted on three male participants. The sex of participants was based on self-report. As our participant sample included only a single sex, we did not perform any sex-based analyses. We did not assess their gender, as we did not anticipate an effect of gender on our study outcomes.
Reporting on race, ethnicity, or other socially relevant groupings	We did not assess the race, ethnicity, or any other socially relevant grouping that our participants may or may not belong to as we did not anticipate an effect of these variables on our study outcomes.
Population characteristics	Participant P2 sustained a C5 motor/ C6 sensory ASIA B spinal cord injury ten years prior to the time of implant. He was between 25-30 years old at the time of implant and between 30-35 years old during the data collection for this study. Participant P3 was of similar age as P2 during implant and data collection and sustained a C6 ASIA B spinal cord injury 12 years prior to implantation. Participant C1 sustained a C4 ASIA D spinal cord injury 35 year prior to implantation. He was 55-60 years old at the time of implant and during data collection.
Recruitment	Participants were recruited through voluntary research registries for people with spinal cord injury.
Ethics oversight	The study was conducted under an Investigational Device Exemption from the U.S. Food and Drug Administration and ethically approved by the Institutional Review Boards at the University of Pittsburgh and the University of Chicago.

Note that full information on the approval of the study protocol must also be provided in the manuscript.

Field-specific reporting

Please select the one below that is the best fit for your research. If you are not sure, read the appropriate sections before making your selection.

[x] Life sciences [ ] Behavioural & social sciences [ ] Ecological, evolutionary & environmental sciences

For a reference copy of the document with all sections, see nature.com/documents/nr-reporting-summary-flat.pdf

Life sciences study design

All studies must disclose on these points even when the disclosure is negative.

Sample size	This study includes three human research participants. Sample sizes for intracortical Brain-Computer Interface (BCI) studies in humans are limited due to the necessity of surgery and limited size of the eligible population.
Data exclusions	No data was excluded from analysis.
Replication	Experiments were replicated in three participants across two study sites. A subset of the results did not hold for one of the three participants, which is likely due to a difference in task performance, as discussed in the manuscript.
Randomization	The target object for each sensation-object mapping trial was randomized per trial. In addition, the mapping from stimulation parameters to cursor coordinates on the participant interface were randomized each trial.
Blinding	The participants were not aware of which cursor coordinate corresponded to which stimulus parameter. They had to make their design choices purely on how the resulting sensation felt, rather than the visual coordinates of the cursor on the interface. The experimenter did know the exact stimulation parameters during the data collection as they needed to monitor whether stimulation was correctly delivered. All participants completed the same experimental conditions in this within-subjects experimental design.

Reporting for specific materials, systems and methods

We require information from authors about some types of materials, experimental systems and methods used in many studies. Here, indicate whether each material, system or method listed is relevant to your study. If you are not sure if a list item applies to your research, read the appropriate section before selecting a response.

Materials & experimental systems

n/a	Involved in the study
☒	☐ Antibodies
☒	☐ Eukaryotic cell lines
☒	☐ Palaeontology and archaeology
☒	☐ Animals and other organisms
☐	☒ Clinical data
☒	☐ Dual use research of concern
☒	☐ Plants

Methods

n/a	Involved in the study
☒	☐ ChIP-seq
☒	☐ Flow cytometry
☒	☐ MRI-based neuroimaging

Clinical data

Policy information about clinical studies

All manuscripts should comply with the ICMJEguidelines for publication of clinical research and a completedCONSORT checklist must be included with all submissions.

Clinical trial registration	NCT01894802
Study protocol	Trial protocol is unavailable as it is part of an ongoing study (IDE G130082).
Data collection	Data were collected in research laboratories set up for the BCI trial at the Universities of Chicago and Pittsburgh. Data were collected during the first (P3, C1), and sixth (P2) year after device implantation.
Outcomes	The purpose of this trial is to collect preliminary safety information and demonstrate that intracortical electrode arrays can be used by people with tetraplegia to both control external devices and generate tactile percepts from the paralyzed limbs. This manuscript presents the analysis of data that were collected during the participants involvement in the trial but does not report clinical trial outcomes. Therefore, these results are are tangential to the primary and secondary outcomes of there overall clinical trial. Interim publication has been approved by the DSMB.

Plants

Seed stocks	n.a.
Novel plant genotypes	n.a.
Authentication	n.a.
